# Estimation of Paracetamol and Aceclofenac in Tablet Formulation by Ratio Spectra Derivative Spectroscopy

**DOI:** 10.4103/0250-474X.45403

**Published:** 2008

**Authors:** A. D. Nikam, Sampada S. Pawar, S. V. Gandhi

**Affiliations:** A. I. S. S. M. S. College of Pharmacy, Kennedy Road, Near R. T. O., Pune-411 001, India

**Keywords:** Aceclofenac, paracetamol, ratio spectra derivative spectroscopy

## Abstract

A new sensitive, simple, rapid and precise method for simultaneous estimation of paracetamol and aceclofenac in combined tablet dosage form has been developed. The method is based on ratio derivative spectrophotometry. The amplitude in first derivative of the ratio spectra at 256 nm and 268 nm (minima) were selected to determine paracetamol and aceclofenac in combined formulation. The method showed good linearity, accuracy and reproducibility. Results of analysis were validated statistically and by recovery studies.

Aceclofenac (AFC) is {o-(2,6-dichloroanilino)phenyl}acetate glycolic acid ester with antiinflammatory and analgesic properties^1^. Paracetamol (PAR) chemically is 4-hydroxy acetanilide^2^. Several analytical methods are reported for determination of AFC and PAR in bulk and pharmaceutical dosage formulations as a single component as well as in combination with other drugs. Recently UV spectrophotometric method for simultaneous estimation of aceclofenac and paracetamol in a combined tablet dosage form has been reported^3^. Extensive literature survey revealed that no method is available for simultaneous estimation of AFC and PAR in combined dosage form by ratio spectra derivative spectroscopy^4^–^6^. Aim of present work was to develop simple, economical, reproducible and rapid method for simultaneous estimation of binary drug formulation.

The instrument used in the present study was JASCO double beam UV/Vis spectrophotometer (Model UV-530) with fixed slit width 2 nm connected to a computer with spectra manager software. All weighing were done on electronic balance (Shimadzu AY 120). AFC and PAR were obtained from Concept Pharmaceuticals Pvt. Ltd. and Cipla Ltd., respectively, which were used as such without further purification. All chemicals used in spectrophotometric analysis were of analytical grade. Tablets of Aceroc-P (Wockhardt Ltd.) labeled to contain AFC 100 mg and PAR 500 mg were procured from the local market.

Standard stock solution was prepared by dissolving 50 mg of PAR and AFC separately in 50 ml of methanol to get concentration of 1 mg/ml. Ten ml of stock solutions were further diluted to 100 ml with phosphate buffer (pH-7, prepared as per IP) to get a working standard solution of concentration 100 μg/ml of each drug. For commercial formulation analysis twenty tablets were weighed accurately and powdered. Powder equivalent to 50 mg of PAR was weighed and transferred to 50 ml volumetric flask; in the same flask 40 mg of pure AFC drug was added and dissolved in methanol by shaking the flask for 10 min. The solution was filtered through Whatman filter paper No. 41 and first few ml were rejected. 10 ml of this filtrate was further diluted to 100 ml with phosphate buffer pH-7. Two millilitres of this solution was further diluted to 10 ml to get final concentration of 20 μg/ml of each drug. Spectra was recorded and processed separately to determine concentration of each drug.

Salinas *et al*^7^developed a ratio spectra derivative spectroscopic method based on dividing the spectrum for a mixture into the standard spectra for each of the analyses and driving the quotient to obtain a spectrum that is independent of the analyte concentration used as a divisor. The use of standardized spectra as divisors minimizes experimental errors and background noise. Easy measurements on separate peaks, higher values of the analytical signals and no need to work only at zero-crossing points (sometimes co-existing compounds have no maximum or minimum at these wavelengths) are advantages for ratio spectra derivative spectrophotometry in comparison with the zero-crossing derivative spectrophotometry. Also, the presence of a lot of maxima and minima in ratio spectra derivative data was another advantage, since these wavelengths give an opportunity for the determination of these compounds in the presence of other active compounds and excipients that possibly interfered with the assay. Using appropriate dilutions of standard stock solution the two solutions were scanned separately. The ratio spectra of different AFC standards at increasing concentrations was obtained by dividing each with the stored spectrum of the standard solution of PAR (30 μg/ml) by computer aid are shown in fig. 1 (a) and the first derivative of these spectra traced with the interval of Δλ= 13 nm (the influence of Δλ for the first derivative of the ratio spectra was tested to obtain the optimum wavelength interval, Δλ= 13 nm was considered to be suitable) are illustrated in fig. 1 (b). Wavelength 268 nm (minima) was selected for the quantification of AFC in AFC+PAR mixture. The ratio and ratio derivative spectra of the solutions of PAR at different concentrations traced with the interval of Δλ= 13 nm by using the standard spectrum of AFC (10 μg/ml) as divisor by computer aid are demonstrated in fig. 2 (a) and (b), respectively. Wavelength 256 nm (maxima) was selected for the quantification of PAR in AFC+PAR mixture. Measured analytical signals at these wavelengths were proportional to the concentrations of these drugs.

**Fig. 1:**
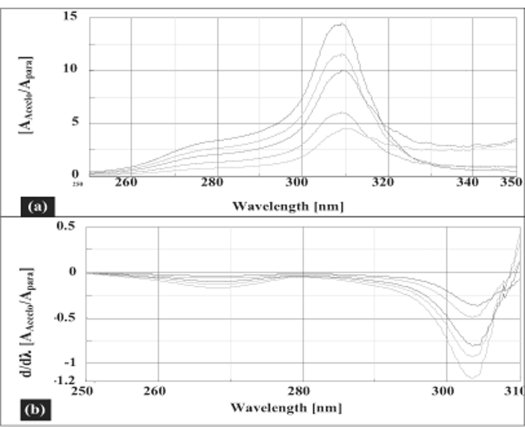
Ratio and first derivative of the ratio spectra AFC and PAR Ratio spectra (a) and first derivative of the ratio spectra (b) of 10-50 μg/ml solution of AFC when 30 μg/ml solution of PAR is used as divisor (Δλ=13 nm).

**Fig. 2:**
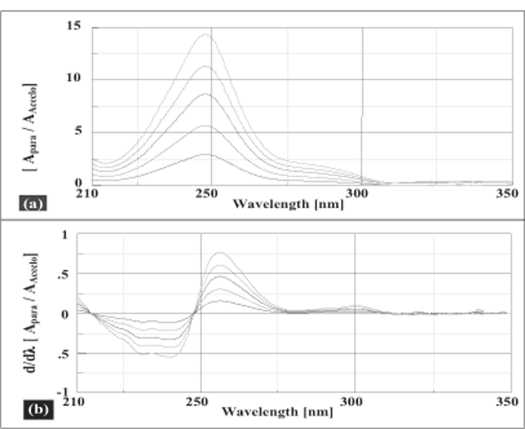
Ratio and first derivative of the ratio spectra PAR and AFC Ratio spectra (a) and first derivative of the ratio spectra (b) of 10 -50 μg/ml solution of PAR when 10 μg/ml solution of AFC is used as divisor (Δλ=13 nm).

Under experimental conditions described linearity was found in the range 10-50 μg/ml with high correlation coefficients for both the drugs. Six replicate readings of drug solution were taken to study the precision of method.% RSD was found to be less than 1.5, indicating reproducibility. For recovery study different volumes of standard solution was added to the fixed volume of sample solution. The total amount of drug added was determined by proposed method. Results of recovery studies are shown in Table 1.

**TABLE 1:** RECOVERY STUDIES OF AFC AND PAR

Level of % Recovery	% Mean Recovery*		Standard Deviation		% RSD		Standard Error	
				
	AFC	PAR	AFC	PAR	AFC	PAR	AFC	PAR
50	100.1	100.06	0.0866	0.07637	0.0865	0.0763	0.0499	0.044
100	100.05	99.97	0.0854	0.08326	0.0853	0.0832	0.0493	0.048
150	99.756	99.986	0.4045	0.12055	0.4054	0.1205	0.2335	0.0395

* Average of three determinations, RSD is relative standard deviation
